# Rapid Mental Workload Detection of Air Traffic Controllers with Three EEG Sensors

**DOI:** 10.3390/s24144577

**Published:** 2024-07-15

**Authors:** Hui Li, Pei Zhu, Quan Shao

**Affiliations:** College of Civil Aviation, Nanjing University of Aeronautics and Astronautics, Nanjing 211106, China

**Keywords:** detection of mental workload, gamma wave, mRMR algorithm, air traffic controller, NASA-TXL

## Abstract

Air traffic controllers’ mental workload significantly impacts their operational efficiency and safety. Detecting their mental workload rapidly and accurately is crucial for preventing aviation accidents. This study introduces a mental workload detection model for controllers based on power spectrum features related to gamma waves. The model selects the feature with the highest classification accuracy, β + θ + α + γ, and utilizes the mRMR (Max-Relevance and Min-Redundancy) algorithm for channel selection. Furthermore, the channels that were less affected by ICA processing were identified, and the reliability of this result was demonstrated by artifact analysis brought about by EMG, ECG, etc. Finally, a model for rapid mental workload detection for controllers was developed and the detection rate for the 34 subjects reached 1, and the accuracy for the remaining subjects was as low as 0.986. In conclusion, we validated the usability of the mRMR algorithm in channel selection and proposed a rapid method for detecting mental workload in air traffic controllers using only three EEG channels. By reducing the number of EEG channels and shortening the data processing time, this approach simplifies equipment application and maintains detection accuracy, enhancing practical usability.

## 1. Introduction

With advancements in science and technology, the human role within human–machine systems is gradually shifting towards higher-level decision-making and monitoring. This shift has led to a reduction in physical labor and an increased mental workload within modern human–machine systems. Research has established that heightened mental workload can lead to rapid fatigue, increased errors, and compromised decision-making, all significant contributors to human-caused accidents. Indeed, air traffic controllers frequently face numerous human–computer interaction tasks within short periods, significantly elevating their mental workload and the likelihood of errors. This has profound implications for operational performance and can even lead to safety incidents in severe cases [1,2]. Given these implications, detecting mental workload at work is of paramount importance. Existing methods for detecting mental workload are primarily categorized into subjective detection methods, objective detection methods, and task-based measures. Subjective detection methods, such as the KSS scale and NASA-TLX scale, are user-friendly and widely applicable but are greatly influenced by subjective factors and exhibit lower accuracy [3,4]. Objective tests, including EEG signal detection, ECG signal detection [5], eye information detection [6], facial feature detection, and voice feature detection, rely on physiological data and can ensure high reliability and rapid status reflection, although they may disrupt actual work or experimental subjects. Among these methods, EEG signals were verified to be reliable for assessing mental workload due to their high temporal resolution, relatively low cost, and portability [7,8,9]. In fact, EEG signals are regarded as the “gold standard” for cognitive status in mental workload detection [10]. Therefore, to achieve both accuracy and rapid detection performance, this paper proposes using EEG signals to assess the mental workload of operators.

Using EEG signals to detect the mental workload of the controllers presents several challenges that need to be addressed: (1) the multi-channel nature of EEG signals increases the computational complexity of the system, potentially leading to dimensionality catastrophe or low classification accuracy [11]; (2) utilizing multiple EEG channels also increases the time required for detection, slowing down the process of mental workload assessment; (3) multi-channel EEG acquisition systems are complex and require specialized technicians for operations such as electrode positioning; and (4) time-consuming steps like Independent Component Analysis (ICA) in EEG data preprocessing can impact the speed of detection, causing delays in mental workload assessment.

Given these challenges, applying multi-channel systems to detect mental workload in practical work scenarios is difficult. To address these issues, a study was conducted to reduce the channels and identify those less affected by ICA processing. This approach not only maintains high accuracy while decreasing the number of channels and detection time, but also enhances convenience by reducing the requirement for applying electroencephalographic cream to multiple electrodes. Overall, the results of this paper are instrumental in facilitating the application of EEG signals for mental workload detection.

The novelty of this paper is evident in three main aspects:

(1) Proposing EEG features associated with gamma waves. The classification results demonstrate that β + θ + α + γ features maintain good robustness across different classification methods, making them better than other features for mental workload detection of controllers.
(2) Introducing the mRMR (Max-Relevance and Min-Redundancy) algorithm for channel selection, with the validity of the results being verified from a neuroscience perspective.
(3) Comparing the changes in detection accuracy before and after Independent Component Analysis (ICA) processing, identifying the channels less affected by ICA processing (CP2, CP3, and CP5), and verifying the reliability of the results by analyzing the range of artifacts removed by ICA processing.

The acronyms used in the text are explained in Table 1 below.

## 2. Related Work

### 2.1. EEG Analysis of Controllers

**Figure 1:**
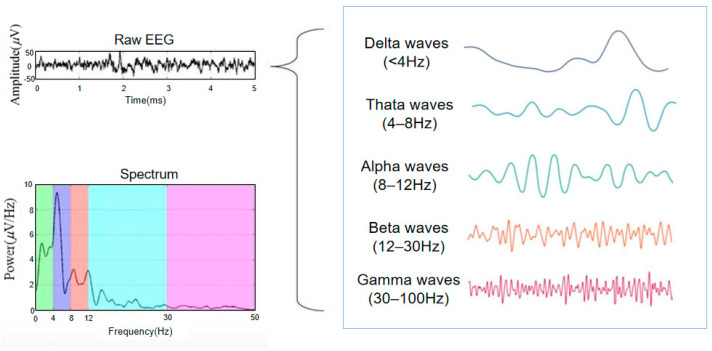
Human brain rhythms: delta wave (1–4 Hz), theta wave (4–8 Hz), alpha wave (8–13 Hz), beta wave (13–30 Hz), and gamma wave (above 30 Hz).

As shown in Figure 1, it is widely acknowledged that human brain waves can be classified into five frequency bands based on different frequencies: delta wave (1–4 Hz), theta wave (4–8 Hz), alpha wave (8–13 Hz), beta wave (13–30 Hz), and gamma wave (above 30 Hz). Different forms of waves can reflect various mental states of the brain. Each type of wave can indicate various mental states of the brain [12,13]. Delta waves are associated with deep relaxation and restorative sleep, while theta waves are prevalent in trance or hypnagogic states. Alpha waves, situated between conscious thought (beta) and subconscious (theta), have a calming effect and promote deeper relaxation and contentment. Beta waves are the most common high-frequency awake waves, while gamma waves represent higher cognitive activity, signaling increased neuronal excitability.

In 2010, Dasari et al. [14] concluded that theta, alpha, and beta waves could be utilized to analyze the shift in the mental state of controllers’ behaviors through EEG examination. Shou et al. [15] discovered that theta waves increased with mental workload based on the EEG data test of the controllers, indicating that theta waves can better reflect the changes in EEG features caused by increasing workload. Thomas et al. [16] analyzed four EEG features using fast Fourier transform: (theta + alpha)/beta; alpha/beta; (theta + alpha)/(alpha + beta); and theta/beta. They found that the content of alpha waves relatively increased and the content of beta waves relatively decreased during severe brain fatigue, while (theta + alpha)/beta increased significantly. Arico et al. [7] assessed the cognitive abilities of the controller trainees by analyzing the frontal and occipital theta waves, as well as parietal theta waves, after a trainee completed simulator tests. Alpha waves were analyzed to verify the reliability of detecting mental workload over time. Puma et al. [17] examined theta and alpha waves and analyzed EEG data in a multitasking environment.

Current research on the detection of air traffic controllers’ mental workload typically focuses on characterizing the four wave bands: delta, theta, alpha, and beta waves, with less consideration of the role of gamma waves in mental state analysis and the prevailing notion that high-frequency signals were considered noise and systematically filtered out in the past, owing to the limitations of computer technology [18]. Although recent technological advances have brought high-frequency brain activity analysis into the mainstream and highlighted the importance of gamma waves in learning, memory, and processing, there remains an analysis of high-frequency brain activity targeted at air traffic controllers.

High-frequency waves are indicative of high-level cognitive activities and are deemed more suitable for analyzing the mental state of air traffic controllers. Given that the prevailing method in current mental workload research involves assessing the sensitivity of EEG features to changes in mental workload and utilizing mathematical algorithms to construct a mental workload detection model [19], this study aims to put forth a range of traditional features and gamma-related features for model development. Our approach involves analyzing both high-frequency and low-frequency signals to pinpoint the most effective feature for mental workload analysis and to establish a robust model.

### 2.2. Channel Selection Techniques

Given the multi-channel nature of EEG signals and its impact on detection speed and usability, numerous scholars have delved into channel selection strategies. In 2015, Alotaiby et al. synthesized the utilization of EEG signal channel selection in classifying mental tasks [20]. Two primary techniques for channel selection in mental tasks are filtering techniques and wrapper techniques. A notable wrapper technique is a channel reduction method based on a subset generation genetic algorithm introduced by Tavakolian et al. [21]. This method employs a random search approach for subset channel selection and utilizes a genetic algorithm to identify the optimal combination of six channels from a pool of 19 channels. The results indicate classification accuracies of 100%, 99.6%, 96.66%, and 88% for subjects 1, 3, 5, 2, and 4, respectively. Amongst the filtering techniques, Lan et al. [22] employed mutual information technology for dimensionality reduction (channel selection and feature projection) and subset channel selection employing a sequential search method of filtering techniques. Utilizing 32 EEG recording channels, the average classification accuracy for all subjects hovered around 80% with seven, ten, and seven channels. Alyasser et al. proposed an EEG-based binary flower pollination algorithm (FPA) and β-climbing method for individual identification channel selection [23], revealing that halving the number of channels can yield high accuracy rates.

In recent years, advancements in channel selection techniques have led to a reduction in the number of channels required for detection tasks. Fu et al. introduced a fatigue detection model based on the Hidden Markov Model and the fusion of physiological and situational knowledge to evaluate the probability of fatigue. By utilizing EEG signals from two channels (O1 and O2) [24] alongside other physiological signals, they achieved a peak accuracy of 92.5%. Li et al. [25] gathered EEG data from 16 channels and computed 12 energy parameters, subsequently reducing the number of electrodes using Kernel Principal Component Analysis (KPCA). Notably, two channels (FP1 and O1) exhibited the highest accuracy of 91.5% in their study. Additionally, Xiong et al. [26] merged Acoustic Emission (AE) and Surface Electromyography (SE) features with a Support Vector Machine (SVM) classifier for driver fatigue detection, attaining a maximum accuracy of 91.3% in a single channel. Jianfeng Hu et al. [27] leveraged entropy measurements to extract features from individual EEG channels. Various entropy metrics were employed to analyze raw EEG signals and enhance performance for a single channel, yielding an impressive accuracy of 96.6%.

In conclusion, channel selection methods encompass filtering and wrapper techniques. Wrapper techniques typically leverage search algorithms and optimization algorithms to identify the optimal subset of channels for classification and detection tasks. Filter techniques reduce the channel count and enhance classification accuracy with fewer channels through dimensionality approximation and channel selection strategies.

Amongst these techniques, mutual information technology holds several advantages over others: 1. independence from prior knowledge [28]: mutual information technology stands out as a reference-free estimation method capable of directly calculating feature correlations using statistical and information theory; 2. all-encompassing consideration of relationships [29]: leveraging mutual information technology permits the robust handling of non-linear relationships by accounting for non-linear dependencies between features, resulting in more precise and comprehensive correlation estimations; and 3. reduced computational burden [30]: compared to intricate algorithms, mutual information techniques entail comparatively lower computational load, facilitating swift channel selection and feature correlation computations.

Therefore, the present study proposes the utilization of a feature selection algorithm rooted in mutual information theory for EEG channel selection. Our objective is to optimize the detection process by employing the minimum number of channels necessary. To achieve this, the selected channels must contain significant mental workload-related information with minimal redundancy. This strategy allows for a reduction in channel count while preserving crucial information, streamlining the detection task with limited channel selection. Specifically, the mRMR (Max-Relevance and Min-Redundancy) algorithm [31] in the mutual information-based feature selection approach highlights essential features by maximizing correlation with target variables and minimizing redundancy. Notably, the mRMR algorithm proves instrumental for channel selection. Consequently, this study proposes the incorporation of the mRMR algorithm for channel selection and validates its efficacy.

## 3. Materials and Methods

### 3.1. Subjects

Given that a majority of participants in the aforementioned experiments were student controllers lacking practical experience, the accuracy of the experimental data was somewhat compromised. To address this limitation, this study recruited 41 air traffic controllers holding control licenses from Nanjing Lukou Airport and Jiangsu Air Traffic Control Bureau. All participants were aged between 26 and 34 years old and provided informed consent prior to their involvement in the research.

### 3.2. Experiment Setup

Due to the inability to manipulate actual traffic flow in a live control setting, the experiment was conducted using a control simulator provided by the Control Simulation Laboratory of Nanjing University of Aeronautics and Astronautics. Participants engaged in control activities within simulated environments with varying levels of traffic density, with the intention of inducing different levels of mental workload by adjusting the complexity of the control scenarios.

In general, traffic represents a primary source of mental workload for air traffic controllers, quantified by the number of aircraft requiring handling within a given timeframe, typically measured in terms of landings and take-offs. Increased traffic volumes require controllers to manage a greater number of aircraft simultaneously, ensuring their safe and efficient operation in both the air and on the ground. This heightened workload involves the allocation of aircraft to appropriate routes, altitudes, and intervals, as well as coordinating and monitoring communications and traffic flow between aircraft. Such responsibilities demand constant vigilance over multiple aircraft and the provision of timely and accurate decisions and instructions. Consequently, heightened traffic flow elevates the mental workload and stress experienced by air traffic controllers.

However, in addition to traffic flow, other special situations can also impact the mental workload of air traffic controllers. These include equipment malfunctions, runway foreign object debris, specific operational demands of airports, and incident management. Such situations are generally referred to as abnormal situations. When special situations occur, air traffic controllers must process information, make decisions, and provide tailored guidance to ensure aviation safety. Feedback from controllers revealed that during abnormal situations, they must consider actions such as resuming flights, avoidance maneuvers, emergency evacuations, and standby protocols to maintain the safe and efficient operation of air traffic. Dealing with such situations elevates their mental workload and exposes them to increased psychological stress.

Based on the aforementioned factors, the mental workload of controllers is typically categorized into three stages: Baseline, High Workload Nominal, and High Workload Off-Nominal [32]. The Baseline stage involves managing a maximum of six aircraft within a specific timeframe, representing a moderate workload that does not involve abnormal scenarios. The High Workload Nominal phase entails handling up to 21 aircraft within a designated timeframe, without encountering abnormal situations. High Workload Off-Nominal encompasses the experimental setup including the exceptional scenarios mentioned earlier, in addition to the High Workload Nominal conditions.

In this study, the abnormal situation pertains to the presence of foreign objects on the runway. When controllers identify such objects, immediate actions must be taken, including promptly notifying the flight tower, issuing warnings and instructions to aircraft during takeoff and landing regarding the foreign objects on the runway, and coordinating closely with the flight tower and ground operations personnel to assess the extent of the hazard. Regular updates on the progress and safety status of foreign object clearance should be provided, ensuring that notifications and instructions are communicated effectively to the flight tower and aircraft.

In addition to the three aforementioned phases, this research incorporates the Overload Nominal scenario to comprehensively evaluate the controller’s mental workload, involving the management of 30 aircraft within a specified timeframe without encountering abnormal situations. Overall, the experimental scenarios in this study are outlined in detail in the following labels in Table 2. The four experimental scenarios correspond respectively to different workload categories, namely Baseline, High Workload Nominal, Overload Nominal, and High Workload Off-Nominal. These labels will be utilized to classify EEG data.

**Figure 2:**
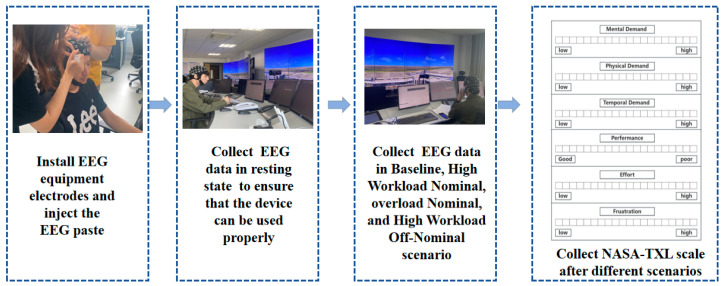
Flowchart of the experiment.

Furthermore, the sequencing of scenarios in this study was determined by the pre-experiment, which involved classifying the EEG data of the controllers into various scenario orders. Based on the pre-experiment results, it was found that the EEG data of the subjects were most distinguishable when presented in the sequence of ‘resting—scenario 4—scenario 3—scenario 1—scenario 2’. Subsequently, the following experiments will adhere to this order. Each scenario had a duration of half an hour, and each subject completed all tasks in the specified sequence: ‘resting—scenario 4—scenario 3—scenario 1—scenario 2’, with a five-minute break between scenarios to complete the NASA-TXL scale assessment. The detailed experimental procedure is outlined in Figure 2. Participants were required to work for over 2 h in a simulated control environment to collect EEG data and provide subjective ratings using the NASA-TXL scale.

### 3.3. Data Process

#### 3.3.1. Feature Extraction

An experiment was conducted in order to conduct a more comprehensive analysis of the mental state of controllers across different scenarios on changes in mental workload. Additionally, a set of detection features was proposed to capture variations in mental workload based on EEG recordings, as illustrated in the table below. Initially, common features such as α, β, δ, θ, θ/β, α/β, and (α + θ)/(α + β) were considered [16,33]. Building upon this foundation, new features related to gamma waves, including γ, γ/(δ + β + θ + α), and γ/(δ + β + θ + α + γ), were introduced. As showing in Table 3, features 1–13 represent traditional mental workload detection features, while features 14–26 pertain to the novel gamma wave-related features proposed in this study.

Before delving into the analysis of the EEG data, various preprocessing steps were carried out on the acquired EEG data, such as eliminating redundant electrodes, re-referencing, filtering, and Independent Component Analysis (ICA).

Then, the EEG signal was filtered with a bandpass between 0.5 and 100 Hz for each subject, followed by wavelet transformation. After completing the preprocessing, we utilized the wavelet transform proposed by Jean Morlet to examine the changes in brain activity of controllers [34]. The wavelet transform of a continuous function x(t) is defined by

(1) $$WT(a,b)=\int_{-\infty }^{+\infty }x(t)\psi_{\left(a,b\right)}(t)dt$$

where

(2) $$\psi_{\left(a,b\right)}(t)=\frac{1}{\sqrt{a}}\psi (\frac{t-b}{a})$$

is the mother wavelet, and a and b are the scale and position of the wavelet, respectively. By translating and dilating the mother wavelet, the wavelet transform achieves exceptional time–frequency localization.

#### 3.3.2. Feature Selection and Channel Selection

After feature extraction, the Support Vector Machine (SVM) classifier is employed to classify the feature data according to different classifications [35]. The study considers four workload classification categories: resting/working, 0/low–medium/high load, 0/low/medium–high load, and 0/low/medium/high load.

For the classification method of resting/working, the EEG data of a controller at rest is labeled as “resting”, while the EEG data of a controller at work is labeled as “working”. The standard Support Vector Machine (SVM) is suitable for binary classification tasks, suitable for the resting/working classification.

For multi-task classification, this study extends the standard support vector machine to multi-task classification using the One-vs-One method [36]. Initially, the labels corresponding to the EEG feature data are assigned based on the workload labels of each scenario mentioned above. Subsequently, the categories are established according to these workload labels. A binary classifier is created for each category to compare it with the other categories individually, resulting in multiple binary classifiers in the end. The various classifications of multitasking are explained below.

Under this classification of ‘0/medium-low/high load’, the feature data of controllers at rest corresponds to the label ‘0 load’, while the feature data at scenario 1 and scenario 2 corresponds to the label ‘medium-low load’, and the feature data at scenario 3 and scenario 4 corresponds to the label ‘high load’.

Under the classification of ‘0/low/medium-high load’, the feature data of the controller at rest corresponds to the label ‘0 load’, while the feature data of the controller in scenarios 2, 3, and 4 correspond to ‘medium-high load’, and the feature data of the controller in scenario 1 corresponds to the label ‘low load’.

Under the classification of ‘0/low/medium/high load’, the controller’s feature data in scenario 1 corresponds to the label ‘low load’, the feature data in scenario 2 corresponds to the label ‘medium load’, and the feature data in scenarios 3 and 4 correspond to ‘high load’.

For each feature, the corresponding feature data are classified by SVM classifier according to the aforementioned method. Additionally, a grid search method is applied to enhance the classification results, identifying the optimal parameter combination for each feature under different classification methods. Subsequently, the best-performing feature is selected based on the classification accuracy across different categories for mental workload detection of the controllers.

Following the selection of the feature with the highest classification accuracy, the study utilizes the mRMR feature selection algorithm to identify the best channel or combination of channels for each subject, based on the rationale explained in Section 2.2. mRMR is a feature selection algorithm that combines correlation and redundancy between features to identify the most correlated features while minimizing redundancy [31]. Among them, the maximum relevance criterion is defined as follows:

(3) $$\max D(S,c),D=\frac{1}{\left|S\right|}\sum_{x_{i}\in S}I(x_{i};c)$$

Here, S represents the set of channels, and c represents mental workload, with $x_{i}$ being one of the channels. The minimum redundancy criterion is described as follows:

(4) $$\min R(S)=\frac{1}{{\left|S\right|}^{2}}\sum_{x_{i},x_{j}\in S}I(x_{i},x_{j})$$

The integration of the aforementioned constraints is termed “minimal-redundancy-maximal-relevance” (mRMR).

(5) maxφ(D,R),φ=D−R

Within this procedure, all channels are treated as a subset of features. The outcome of this process is a reduced number of channels containing substantial mental workload-related information with minimal redundancy between channels.

Initially, the subset comprises 59 channels after eliminating irrelevant ones. Subsequently, each channel’s data are assessed based on its correlation with mental workload. Channels are then iteratively included or excluded to ensure that the selected channel combination exhibits a strong correlation with workload and minimal redundancy among the channels. By retaining channels with high-performance correlation and eliminating those with low correlation, a reduction in the number of channels can be achieved while upholding detection accuracy and circumventing accuracy degradation resulting from channel elimination.

### 3.4. Model and Optimization

**Figure 3:**
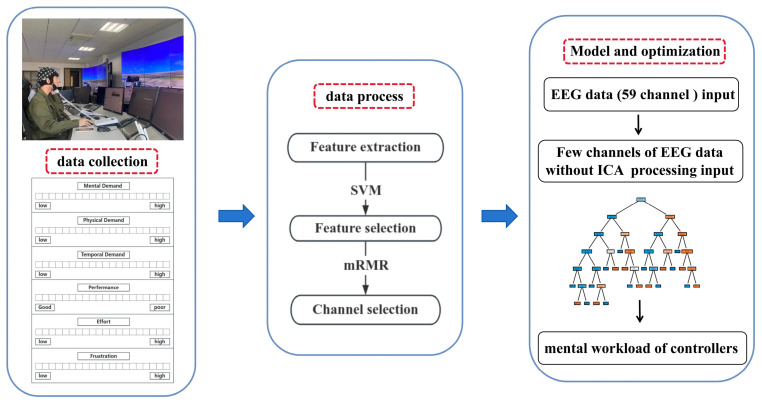
After collecting the data, the data were processed, and the feature was selected according to classification accuracy. Based on the feature data, the metal workload detection model was developed and optimized.

Firstly, a mental workload detection model will be established based on the data from all channels of the feature with the highest classification accuracy. This model is further optimized by inputting the data from selected channels processed by Independent Component Analysis (ICA) and those not subject to ICA processing. Subsequently, channels less impacted by ICA processing are identified, and data from these channels are utilized as input for the model. Through these steps, the optimized model not only reduces the number of channels but also eliminates the need for the ICA processing step. The detailed workflow of this study is depicted in Figure 3.

## 4. Results

### 4.1. NASA-TXL Data

The NASA-TXL Scale (NASA Task Load Index) is a multidimensional measure of workload across six dimensions: Mental Demands, Physical Demands, Temporal Demands, Own Performance, Effort, and Frustration. In this paper, the NASA-TXL scale [37] was utilized to evaluate controllers’ perceptions of mental workload.

**Figure 4:**
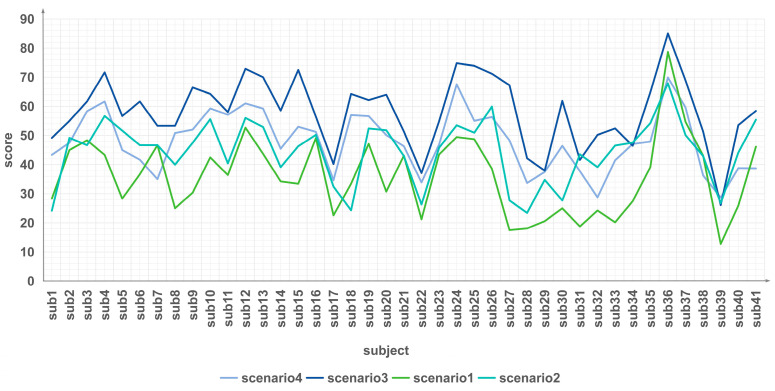
After completing the task corresponding to each scenario, the subjects filled in the NASA-TXL scale. The figure above displays the results of the NASA-TXL scale filled in by 41 subjects after completing the 4 scenarios.

A total of 41 participants assessed the NASA-TXL scale across four scenarios, with findings presented in Figure 4. While individual subjects exhibited slight variations in workload perception for each scenario, the overall trend remained consistent: the Overload Nominal scenario elicited the highest mental workload among all subjects, while the Baseline scenario recorded the lowest cognitive load. Notably, the High Workload Off-Nominal scenario consistently resulted in slightly higher workload compared to the High Workload Nominal scenario.

These outcomes indicate that the control scenarios outlined in this study effectively induce varying levels of mental workload among controllers.

### 4.2. Classification Accuracy of All Features

**Figure 5:**
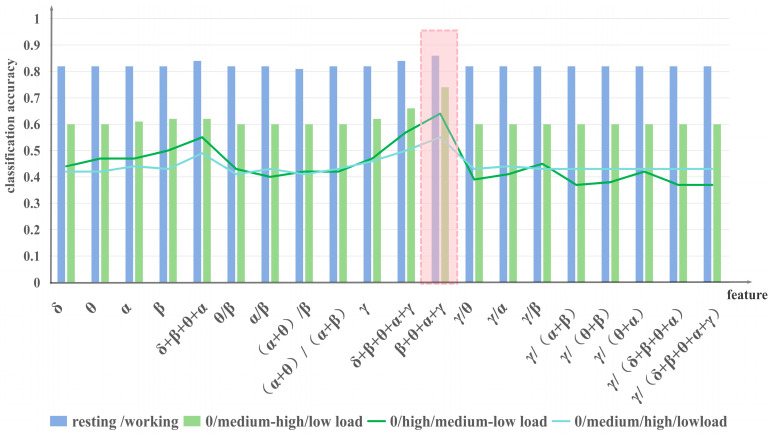
According to the workload variations scenario, we identified four classifications. The classification results can be observed in the figure above. It can be seen that the classification accuracy of β + θ + α + γ is the highest regardless of the classification method.

The classification accuracy results for all features are illustrated in Figure 5. The accuracy of all features surpasses 0.8 in the resting/working classification, indicating the feasibility of data classification and the validity of the proposed features. Notably, the feature β + θ + α + γ demonstrates the highest classification accuracy at 0.86 and outperforms other features across all classification methods, showcasing its robustness across different classification categories. Consequently, the mental workload detection model to be established in the subsequent section will be based on this feature.

The classification outcomes in this study align with existing research [38], highlighting the efficacy of utilizing combined frequency rhythms over individual rhythms for classification purposes. Moreover, the EEG features related to gamma waves introduced in this study exhibit superior suitability for detecting mental workload in controllers compared to conventional features.

### 4.3. Full-Channel Mental Workload Detection Model

The Decision Tree Classification Model was selected for workload detection due to its suitability for small sample sizes. Construction of the model involved utilizing the feature data of β + θ + α + γ from 41 subjects and their corresponding mental workload labels.

During the analysis, the data were randomly split into training and validation sets, with a maximum tree depth of 10 set to prevent overfitting. Leave-one-out cross-validation (LOOCV) was employed [39], where each subject’s sample served as the validation set in a rotating fashion. This approach ensures that each subject’s sample is validated and provides a more precise evaluation of the model’s performance.

Key performance measures in our analysis include accuracy, precision, recall, and the F1-score, which collectively assess the model’s predictive capability. Accuracy represents the proportion of correctly classified samples, while precision is the ratio of correctly predicted positive samples to all predicted positive samples. Recall measures the proportion of correctly predicted positive samples out of all true positive samples. The F1-score combines both recall and precision, calculated as the harmonic mean of the two [40,41].

To optimize the model’s accuracy, we employed a grid search method to identify the best parameter combinations, a widely recognized technique in the field of machine learning for parameter tuning [42]. This method thoroughly tests all parameter combinations within a specified range to determine the most effective settings for the model, known for its robustness.

**Figure 6:**
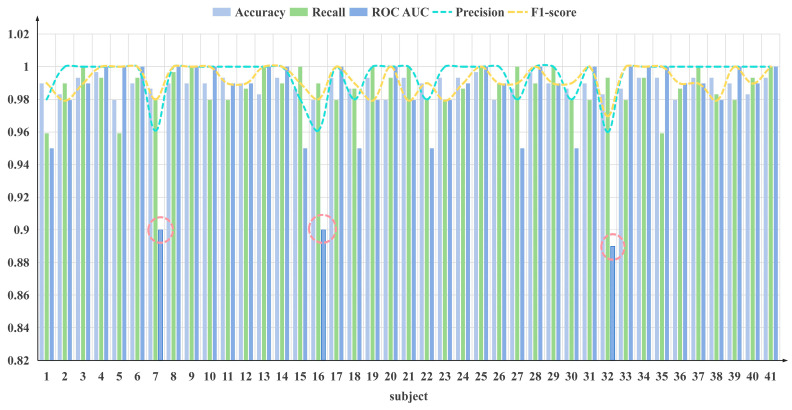
The figure above illustrates the changes in the various evaluation features of the model after inputting EEG data from different subjects. Among them, subjects 7, 17, and 32 correspond to poorer detection results.

The modeling results are shown in Figure 6, in the evaluation of the model’s performance, the accuracy for the 41 subjects ranged from 0.996 to 0.979, with a fluctuation of 0.9875 ± 0.0085. This demonstrates that the model’s accuracy meets the required standard for practical application in workload detection for controllers.

Regarding precision, the values were predominantly high, ranging from 0.96 to 1, indicating the model’s high accuracy. Additionally, both recall and F1-score values aligned with our expectations, with recall displaying values of 0.9795 ± 0.0205 and F1-score reaching as high as 0.969.

However, the ROC AUC of the model did not consistently perform as well as the aforementioned features. Notably, the ROC AUC values for subjects 7, 16, and 32 exhibited superior performance, reaching up to 0.95 to 1, indicating stronger predictive capabilities for most subjects. Conversely, the features for subjects 7, 16, and 32 displayed lower performance, ranging from 0.889 to 0.90, as shown in the Figure above, suggesting relatively poor predictive performance for these subjects.

While the workload model performed as anticipated for most subjects and could be applied for their workload detection, the performance of features for subjects 7, 17, and 32 was relatively subpar. This implies that the workload detection model may not be optimal for these three subjects. However, in practical applications, the detection accuracy for these subjects was deemed sufficient, indicating applicability for most subjects. Despite suboptimal performance for a few subjects, the overall detection accuracy meets the necessary criteria for practical implementation.

### 4.4. Channel Selection mRMR Algorithm

From the above analysis, it is evident that feature β + θ + α + γ exhibits the highest accuracy among different classification categories. Therefore, the next phase of this research paper will focus on identifying the optimal channel combination for each subject based on the feature β + θ + α + γ. The mRMR algorithm, a feature selection technique that emphasizes maximizing the correlation with the target variable and minimizing redundancy among features, will be utilized for this purpose [31].

**Figure 7:**
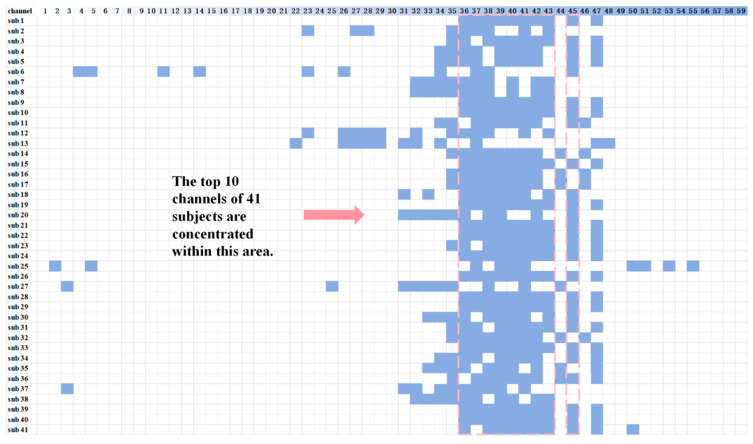
The number of features was set to 10 (the 10 best channels corresponding to each subject), and the results are shown above. It can be seen that the optimal channel combination for the 41 subjects is not exactly the same. However, the distribution of the better-performing channels is concentrated.

To identify channels most relevant to mental workload, we applied the mRMR algorithm to our original set of 59 channels, with the target variable being mental workload. Figure 7 displays the results of the channel screening, revealing the top 10 ranked channels for the 41 subjects. Notably, the optimal channel varied across subjects, indicating that there is no universal set of channels that is optimal for all subjects.

However, it is worth noting that channels 38, 39, 40, and 41 appeared frequently across subjects, suggesting that while a universally optimal channel combination may not exist, it is feasible to identify channel combinations that perform well for the majority of subjects.

**Figure 8:**
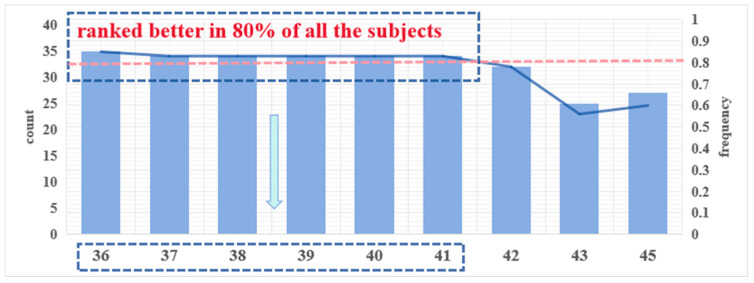
Counting the number and frequency of occurrences of the better-performing channels, it can be seen that channels 36–41 occur more frequently. These channels outperform others in over 80% of the subjects.

Based on the results of the mRMR algorithm for the 41 subjects, Figure 8 below showcases the top 10 channels, where channels CP2 (channel 36), CP3 (channel 37), CP4 (channel 38), CP5 (channel 39), CP6 (channel 40), and TP7 (channel 41) appeared in more than 80% of the subjects.

This underscores the challenge of achieving high-accuracy mental workload detection for all subjects using a single channel. Therefore, to mitigate variability and improve model applicability across subjects, expanding the number of channels used is essential. Incorporating data from channels with high-frequency occurrences as model inputs can aid in assessing mental workload in controllers. Given the recurrent appearance of CP2, CP3, CP4, CP5, CP6, and TP7, we will delve into the analysis of these channels.

### 4.5. Reasons Why Selected Channels Can Be Used for Mental Workload Detection

According to the spatial location, the human cerebral cortex is divided into four lobes: frontal, parietal, temporal, and occipital. Each lobe is associated with a specific letter and number: F for Frontal, T for Temporal, P for Parietal, O for Occipital, and C for Central. While the term “Central” does not correspond to an actual brain region, it is used to aid in the identification of typical EEG activity.

The aforementioned channels CP2, CP3, CP4, CP5, CP6, and TP7 are primarily concentrated in the parietal lobe with partial involvement in the temporal lobe. The parietal lobe, located behind the central sulcus, plays a key role in processing spatial information and integrating various types of sensory input, including visual and auditory information [43,44,45,46,47]. On the other hand, the temporal lobe, positioned on both sides of the brain and adjacent to the ears, is responsible for auditory processing, memory functions, and features specialized regions on its outer surface for speech comprehension [48,49,50,51,52].

**Figure 9:**
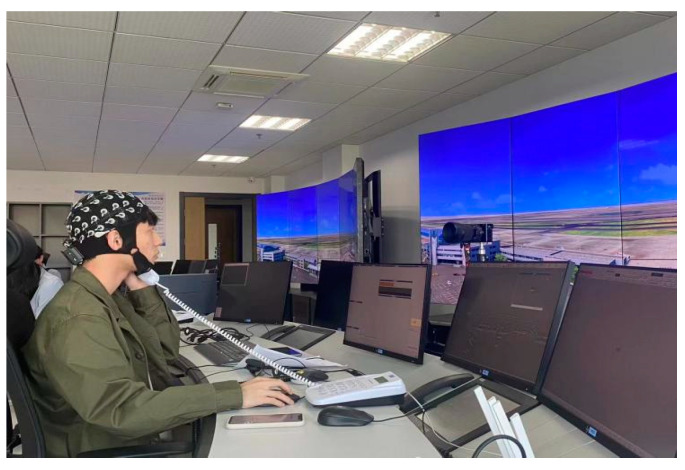
Controller at work.

As illustrated in Figure 9, the work of controllers involves simultaneous monitoring of control screens, external views, communication with the captain, and traffic direction. Consequently, they need to integrate information from visual and auditory sources. Therefore, detecting the mental workload of controllers through electrodes CP2, CP3, CP4, CP5, and CP6 in the central parietal area is feasible. Additionally, since activities such as answering phone calls and communication require language comprehension, detecting the mental workload of controllers through channel TP7 is also plausible. In summary, the channels CP2, CP3, CP4, CP5, CP6, and TP7 identified in this study using the mRMR algorithm can be utilized to assess the cognitive load of controllers.

### 4.6. Channels Less Effected by ICA

Efficient and accurate detection of mental workload based on EEG signals also hinges on solving the challenge of signal preprocessing. The EEG data preprocessing steps employed in this study, which involve the elimination of unnecessary electrodes, re-referencing, and filtering, demonstrate high efficiency without compromising detection speed. However, the Independent Component Analysis (ICA) step is relatively time-consuming, taking about half an hour to process a 30-minute segment of EEG data. Thus, optimizing this process is crucial for swiftly detecting the mental workload of controllers.

During their work, controllers are required to answer phone calls and use the mouse, resulting in various physiological artifacts such as muscle activity (EMG), eye movements or flickering (EOG), and heart activity (ECG). The primary objective of employing independent component analysis (ICA) is to eliminate these artifacts and enhance the quality of EEG signal measurements by minimizing the impact of contaminated signals on genuine EEG data [53]. Presently, the application of ICA to multi-channel EEG is beneficial in segregating independent brain and non-brain contributions within the recorded mixtures [54]. Nevertheless, it is important to note that ICA also carries potential drawbacks, as it may inadvertently result in the loss of valuable information while eliminating noise [55].

Therefore, this study aims to identify EEG channels that are less susceptible to artifacts. By utilizing these channels to assess the mental workload of controllers, there is no requirement to account for the impact of contaminated signals on the actual EEG data. Subsequently, the need for the Independent Component Analysis (ICA) process for artifact removal is greatly diminished, resulting in a significant reduction in data preprocessing time. This streamlined approach alleviates concerns about potential loss of valuable information associated with ICA processing.

In this study, the average detection accuracy of the channels processed without ICA is compared to those processed with ICA to identify the EEG channels less affected by artifacts. Channels exhibiting minimal disparity in detection accuracy between ICA-processed and non-ICA-processed data are considered less susceptible to artifacts and suitable for mental workload detection without the use of ICA processing.

**Figure 10:**
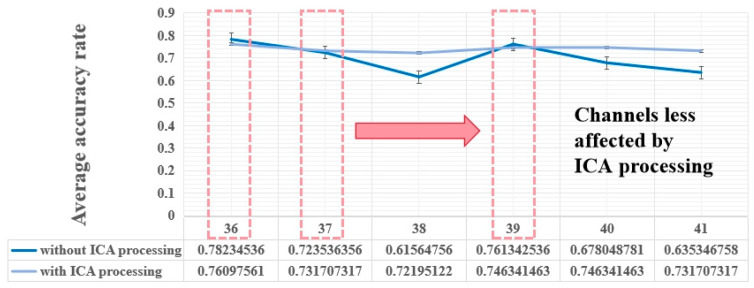
The figure above illustrates the impact of six channels with or without ICA processing on the average detection accuracy. It is evident that the presence or absence of ICA processing has a minimal effect on the average detection accuracy of channels 36, 37, and 39. Furthermore, the utilization of ICA processing does not significantly affect the detection accuracy of these channels.

To assess the impact of artifacts on the aforementioned channels, data from channels CP2, CP3, CP4, CP5, CP6, and TP7 were fed into the model both with and without ICA processing. The outcomes are depicted in Figure 10, revealing that channels CP2, CP3, and CP5 are minimally affected by ICA processing, with no significant impact on their detection results.

**Figure 11:**
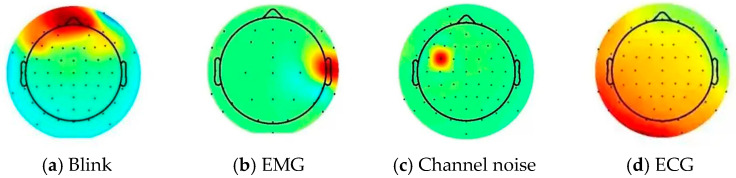
The figure above illustrates the distribution of the four types of typical noise components that are mainly processed by ICA processing.

To validate these findings, a literature review was conducted. Independent component analysis (ICA) has emerged as a widely utilized tool in biomedical signal processing, particularly for eliminating interfering signals [56]. It primarily targets four common noise components [57,58]:

(1) Blink Interference: the topography of the independent components is mainly distributed in the frontal eye electrodes, as shown in Figure 11a.

(2) EMG: the topography of the independent component is mainly concentrated in the left/right temporal lobes, as shown in Figure 11b.

(3) Channel noise: Mainly caused by poor contact between the channel and the scalp during acquisition. It is typically characterized by a concentration on one electrode in the topography, as shown in Figure 11c.

(4) The most obvious feature of ECG is the time-domain information of this component, which can be observed from the signal of this component with obvious ECG QRS wave, as depicted in Figure 11d.

**Figure 12:**
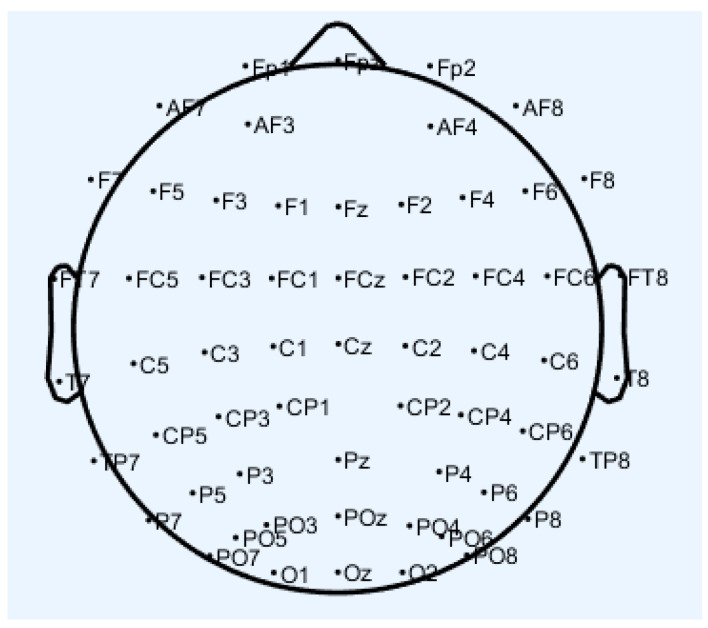
The figure above illustrates the distribution of channel locations for the EEG devices used in this paper.

Upon comparing these noise components with the electrode maps in Figure 12 of the EEG device utilized in this study, it is evident that channels CP2, CP3, and CP5 are unaffected by blinks and EMG interference. Meanwhile, since the most obvious feature of ECG is the time-domain information rather than the frequency domain feature β + θ + α + γ used in the detection in this paper, the signals of these channels are also not affected by ECG. Moreover, the channels remain unimpacted by channel noise, as the EEG equipment used in this research is adept at detecting poor scalp contact during data acquisition.

In conclusion, the signals from channels CP2, CP3, and CP5 exhibit minimal susceptibility to artifacts and can be leveraged for mental workload detection without the necessity of ICA processing. Subsequently, these channels will undergo further analysis in the study.

### 4.7. Rapid Mental Workload Detection Model

Our initial approach involved assessing controllers’ mental workload using individual channels (CP2, CP3, and CP5). Across all 41 participants, single-channel detection yielded significantly lower accuracy compared to full-channel detection, exhibiting considerable instability. This outcome aligns with the prior observation that mental workload detection across all subjects proved unattainable using just one channel. While some subjects achieved single-channel detection accuracy exceeding 0.8, others experienced notably lower precision at 0.4. This variation underscores the influence of inter-individual differences, rendering certain subjects unsuitable for analysis regardless of the channel utilized.

Given the inferior accuracy of single-channel detection compared to full channels, it is plausible that the limited information within a single channel hinders effective mental workload detection in controllers. To enhance detection stability and broaden applicability across subjects, augmenting the number of channels is imperative. This strategy not only enriches the informational content across channels but also diminishes inter-individual variability.

Subsequently, we combined the three selected single channels (CP2, CP3, and CP5) into three pairs, generating corresponding channel combinations for input into the model to ascertain detection accuracy. The results revealed superior performance of dual channels compared to single channels in terms of both accuracy and stability. Notably, most subjects achieved accuracy levels up to 0.9 across all channel combinations. Following exclusion of poorly correlated channels, some subjects even attained perfect accuracy. However, the stability of dual-channel performance varied, with certain subjects displaying incompatibility with the model regardless of the chosen combination. For instance, channels 36 and 37 exhibited a detection accuracy of only up to 0.872, falling short of the desired accuracy threshold. Each dual-channel combination was associated with one to four subjects displaying low detection accuracy.

In order to further improve the accuracy and practicality of the model and address the issue of unsuitability for certain individuals, it is crucial to continue increasing the number of channels. This will ensure consistent detection accuracy across different subjects. In cases where two-channel data are insufficient for stability, this study utilizes three-channel data to detect the mental workload of controllers. The corresponding feature data from channels CP2, CP3, and CP5 of 41 subjects is inputted into the model. Compared to the two-channel detection, the performance of the three-channel detection is significantly improved, with 34 subjects being correctly detected up to 1 and a detection accuracy as low as 0.986. The three-channel model achieved high accuracy for all subjects.

### 4.8. Verification of the Selected Channels

The higher accuracy of the three-channel detection compared to the full-channel detection confirms the effectiveness of our method of channel selection using the mRMR algorithm. This demonstrates that the algorithm can remove redundant channels while retaining informative and relatively independent ones.

Moreover, for these 41 subjects, the three-channel model presented in this study can be directly applied in their work settings. Only channels 36, 37, and 39 need to be processed when positioning the electrodes and applying EEG paste. Furthermore, the corresponding mental workload can be determined by calculating the values of the features β + θ + α + γ in these three channels and inputting them into the model.

This technique significantly enhances the timeliness and relevance of the test while maintaining its accuracy, aiding in the rapid detection of mental workload in aerospace mission operators.

## 5. Discussion

In this paper, the best-performing feature γ + β + θ + α was selected to establish a mental workload detection model for controllers based on full channels. Additionally, the model was optimized through channel reduction to improve its practicality. The results indicate that rapid detection of controller mental workload can be achieved using only three channels.

### 5.1. Feature Extraction

As features directly impact the accuracy of the detection, it is important to initially propose features with better performance. Previous studies often treat EEG raw signals larger than 50 V as artifacts [59,60], with limited research on gamma waves. However, in 2016, Thiago L.T. detected drowsiness based on spectral power features γ/δ and (γ + β)/(δ + α) with better results [61]. Despite fewer studies considering high-frequency gamma waves in the selection of fatigue and workload features, these waves were shown to be related to work and memory. Hence, it is reasonable to consider features associated with gamma waves for workload detection.

To address the research gap in workload detection for controllers, this study first proposes a set of power spectral features encompassing 13 common workload detection features related to δ, α, β, and θ waves, along with 13 new features associated with gamma waves.

Upon proposing and evaluating the features, it was found that among the 26 features, the two best-performing ones are associated with gamma waves. Additionally, the assessment of the absolute energy of the five bands (γ, β, α, θ, δ) aligns with previous literature on the absolute energy of delta, theta, alpha, and beta waves [62].

Furthermore, the study drew additional conclusions that are in line with its findings: (1) The evaluation result of the feature β + θ + α + γ is superior to that of β + θ + α + γ + δ, signifying a decline in performance with the addition of δ. It is notable that the δ wave is not suitable for mental workload detection, consistent with research indicating its prominence during mental trance or hypnotic states in individuals [63,64,65], rather than during the waking state of a controller at work; and (2) the evaluation result of β + θ + α + γ + δ is better than that of δ + β + θ + α, indicating enhanced performance with the addition of γ, aligning with the expectation that features related to gamma waves can be utilized for workload detection. Based on this conclusion, future research can focus on selecting features related to gamma waves, exploring their mechanism in detecting mental workload, and investigating additional relevant and high-precision features.

### 5.2. Feature Selection

Following the proposal of features, it is essential to compare and determine the most suitable one for the corresponding testing work. Li Wei [25] calculated 12 energy features based on EEG data and utilized the Grey Relational Analysis (GRA) method to identify the most effective feature for detecting driver fatigue. While GRA can comprehensively consider the effects of multiple factors, it requires data normalization, increasing computational complexity, and involves subjective judgments in determining weighting parameters and the calculation method of grey correlation, which could potentially impact the final outcomes. Additionally, Thiago L.T [61] employed the Wilcoxon signed-rank test to evaluate the validity of γ/δ and (γ + β)/(δ + α)in detecting sleepiness, a method similar to the approach discussed in this paper, involving a comparison of new features with existing ones to confirm their reliability. However, this method solely evaluates feature quality by identifying significant changes without providing a quantitative assessment of the features.

In this study, a Support Vector Machine (SVM) classifier is employed to categorize feature data using various classification methods, eliminating subjectivity associated with grey correlation analysis and allowing for a quantitative evaluation of feature performance based on classification accuracy. This approach facilitates the identification of the optimal feature for model development compared to the aforementioned methods.

### 5.3. Channel Selection Techniques

The abundance of EEG channels can significantly impact EEG’s application and popularity, necessitating the screening of channels to retain effective ones while discarding those that are not useful. Li Wei [25] utilized Kernel Principal Component Analysis (KPCA) to reduce the number of electrodes and developed a driver fatigue evaluation model using regression equations based on EEG data from two significant electrodes (Fp1 and O1). Selecting the appropriate kernel function and related parameters in KPCA is crucial, as different choices lead to varied downscaling effects, with no universally optimal method for determining parameters requiring experimental adjustment. Min proposed a simplified approach [66] for electrode selection, calculating electrode weights based on accuracy to identify the most crucial electrodes, which may prove ineffective when two-channel detection accuracy falls short.

To address this issue, we introduced the mRMR algorithm for channel selection and validated its effectiveness. This method avoids the need for selecting kernel functions and parameters and allows for the arbitrary selection of channels based on demand. Moreover, the channel combinations identified by this algorithm demonstrate a strong correlation with workload and are relatively independent of each other.

### 5.4. Comparison with Existing Detection Methods

In this study, a full-channel controller workload detection model was established based on EEG data, achieving model accuracy ranging from 0.979 to 0.996 for our 41 subjects. Although the ROC AUC value for subject 33 did not reach 0.889, the overall results of the full-channel model met expectations and could be applied effectively for detecting controllers’ workload.

Given the scarcity of existing mental workload detection models for controllers, this study compares with a fatigue detection model. Nan Wu et al. [67] proposed a speech-based fatigue state detection method that integrates the Quantum Genetic algorithm with an adaptive strategy, achieving a detection accuracy of up to 98.5%. Zhang et al. [68] utilized wavelet entropy and spectral entropy (SE) of EEG, as well as wavelet entropy of EOG and amplitude entropy (AE) of EMG to estimate driving fatigue levels, achieving accuracies ranging from 96.5% to 99.5%. Compared to existing models, this paper exhibits a notable advancement in accuracy. Additionally, the superior accuracy achieved may be attributed to the higher precision of EEG data [69] compared to speech and EMG data.

### 5.5. Comparison with Existing Research

Prior studies have generally improved detection speed by reducing channel numbers and developing channel-less models. Among existing channel-less models, Fu et al. introduced a fatigue detection model based on the Hidden Markov Model and the fusion of physiological and situational knowledge to assess fatigue probability. By using EEG signals from two channels (O1 and O2) [24] and other physiological signals, the model attained a peak accuracy of 92.5%. Li et al. [25] gathered EEG data from 16 channels and computed 12 energy parameters. Employing Kernel Principal Component Analysis (KPCA) to trim electrodes, the study achieved the highest accuracy of 91.5% with two channels (FP1 and O1) in experimental data. Xiong et al. [26] combined Acoustic Emission (AE) and Surface Electromyography (SE) features with a Support Vector Machine (SVM) classifier for driver fatigue detection, securing the top accuracy of 91.3% in the P3 channel. Jianfeng Hu et al. [27] utilized entropy measurements to extract features from individual EEG channels. Sample entropy (SE), fuzzy sample entropy (FE), fuzzy entropy (AE), approximate entropy, and spectral entropy (PE) were employed to analyze raw EEG signals, optimizing performance for a single channel with the highest accuracy reaching 96.6%.

The comparison presented in this paper demonstrates that the method, while deviating slightly from current technology in terms of channel quantity, has achieved a significant advancement in model accuracy with fewer channels. This breakthrough not only maintains or even surpasses the accuracy of multi-channel detection but also reduces the number of channels. Additionally, the paper identifies channels less affected by Independent Component Analysis (ICA) processing, reducing data preprocessing time and facilitating rapid mental workload detection.

### 5.6. Influence of the Number of Channels on Detection Accuracy

**Figure 13:**
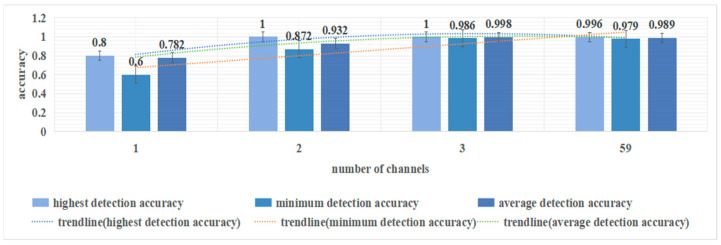
The figure above illustrates the variation in maximum detection accuracy, minimum detection accuracy, and average detection accuracy for different numbers of channels (the channel or combination of channels with the highest average detection rate at that specific number of channels).

Single-channel, two-channel, three-channel, and all-channel data are employed to detect workload, each yielding various outcomes as showing in Figure 13. While all-channel data offer higher stability and accuracy, processing multiple channels can impact detection speed. Therefore, the aim is to minimize the number of channels while maintaining stability and accuracy. In this study, single-channel detection exhibits the lowest accuracy and stability, failing to achieve high detection accuracy across all subjects. Dual-channel outperforms single-channel, but further enhancement is required. The research findings indicate that three channels can achieve rapid and accurate detection of controller mental workload.

It is essential to note that, in practical detection work, a lower number of channels may result in unstable performance, while an excessive number can lead to redundancy and inconvenience. Thus, the number of channels may need to be adjusted based on the specific circumstances. For instance, when the number of subjects is small, two channels may suffice, but for a larger number of subjects, three channels may be required. In this study, three-channel data meet the accuracy and stability requirements for detecting mental workload in the 41 subjects.

### 5.7. Methods for Dealing with Individual Variability

In addition to the existing findings, this study also observed that the optimal channel combinations varied across individual subjects when using the mRMR algorithm for channel selection, indicating inter-individual variability. To address this issue, the study initially identified and combined channels with high occurrence frequencies and then selected the most suitable channel combinations for 41 subjects.

In practical applications, flexibility in usage is warranted based on specific circumstances. For instance, with fewer controllers, EEG data from each controller can be input into the mRMR algorithm to identify the optimal channel for each individual, followed by the development of dual-channel models tailored to each controller to enhance precision and efficiency, avoiding model instability across individuals. For a larger number of controllers, it is advisable to screen channel combinations suitable for all controllers, as demonstrated in this study. If dual-channel setups fail to meet accuracy and stability requirements, increasing the number of channels to establish three-channel models is recommended.

### 5.8. Contributions and Limitations

This study put forward a theoretical feature associated with gamma waves and verified its usability. Additionally, the study demonstrated the feasibility of utilizing the mRMR algorithm for channel selection, enriching the theoretical framework for workload detection models of controllers. The study’s results also facilitate the practical application of EEG equipment for detecting mental workload in controllers, enhancing the technology’s usability.

Practically, the proposed model reduces the number of channels while maintaining accuracy, simplifying the application of EEG-based workload detection and and precise detection of the controller’s mental workload.

However, the study has its limitations. While the usability of β + θ + α + γ was verified, the model parameter settings are tailored to the existing subjects. Their applicability to other subjects still requires verification.

## 6. Conclusions

In an effort to achieve rapid and accurate detection of air traffic controllers’ mental workload, this study explored the mental workload of 41 controllers under four load levels of mental workload with higher accuracy and speed. The findings suggest that:

(1) Compared with traditional features related to delta, theta, alpha, and beta waves, features related to gamma waves, such as β + θ + α + γ, exhibit consistent robustness across diverse classification categories and demonstrate superior performance in the detection of mental workload.
(2) The mRMR (Max-Relevance and Min-Redundancy) algorithm proves effective for channel selection, as confirmed by the validation of the results from a neuroscience perspective.
(3) Due to the subtle variations in EEG signals among subjects, a single-channel detection scheme that applies universally to all 41 subjects in this study is unattainable. Nevertheless, by extracting generalized patterns and common features from multiple individuals’ data, optimal channel combinations adaptable to multiple subjects can be identified, enabling the establishment of a mental workload detection model with fewer channels.
(4) Through the examination of controller data, it was determined that CP2, CP3, and CP5 are less susceptible to artifacts and Independent Component Analysis (ICA) processing. Hence, when using EEG data from these channels for detection, the necessity for ICA processing is eliminated, significantly reducing processing time.

These research findings pave the way for air traffic controllers to detect mental workload with enhanced accuracy and efficiency. The streamlined process of applying EEG equipment in detecting mental workload among controllers not only simplifies the procedure but also accelerates the detection process, thereby enhancing the practical usability of this technology.

## Figures and Tables

**Table 1:** Explanation of acronyms.

Acronyms	Full Name	Usage
mRMR	Max-Relevance and Min-Redundancy	Feature selection
ICA	Independent Component Analysis	Artifacts removal
KSS	Karolinska Sleepiness Scale	Detection of physiological and psychological state
NASA-TXL	NASA Task Load Index	Detection of task loads from six dimensions
SVM	Support Vector Machine	Used for data classification in this paper
KPCA	Kernel Principal Component Analysis	Used for channel selection
EMG	Electromyography	Techniques used to measure the electrical activity of muscles
EEG	Electroencephalography	Techniques for recording the electrical activity of neurons in the cerebral cortex
ECG	Electrocardiogram	Techniques for recording the electrical activity of the heart

**Table 2:** Experimental scenarios.

Scenario	Workload	Air Traffic Flow
Scenario 1	Baseline	6
Scenario 2	High Workload Nominal	21
Scenario 3	Overload Nominal	30
Scenario 4	High Workload Off-Nominal	21

**Table 3:** Features.

Number	Feature	Number	Feature
1	δ	14	γ
2	θ	15	δ + β + θ + α + γ
3	α	16	β + θ + α + γ
4	β	17	γ/δ
5	δ + β + θ + α	18	γ/θ
6	δ/(δ + β + θ + α)	19	γ/α
7	θ/(δ + β + θ + α)	20	γ/β
8	α/(δ + β + θ + α)	21	γ/(α + β)
9	β/(δ + β + θ + α)	22	γ/(θ + β)
10	θ/β	23	γ/(θ + α)
11	α/β	24	γ/(θ + β + α)
12	(α + θ)/β	25	γ/(δ + β + θ + α)
13	(α + θ)/(α + β)	26	γ/(δ + β + θ + α + γ)

## Data Availability

The data presented in this study are available on request from the corresponding author. The data are not publicly available due to confidentiality issues.
